# Transmutation of All German Transuranium under Nuclear Phase Out Conditions – Is This Feasible from Neutronic Point of View?

**DOI:** 10.1371/journal.pone.0145652

**Published:** 2015-12-30

**Authors:** Bruno Merk, Dzianis Litskevich

**Affiliations:** 1 Institute of Resource Ecology, Helmholtz-Zentrum Dresden - Rossendorf, Dresden, Germany; 2 Center for Materials and Structure, University of Liverpool, Liverpool, Merseyside, United Kingdom; Institute for Health & the Environment, UNITED STATES

## Abstract

The German government has decided for the nuclear phase out, but a decision on a strategy for the management of the highly radioactive waste is not defined yet. Partitioning and Transmutation (P&T) could be considered as a technological option for the management of highly radioactive waste, therefore a wide study has been conducted. In the study group objectives for P&T and the boundary conditions of the phase out have been discussed. The fulfillment of the given objectives is analyzed from neutronics point of view using simulations of a molten salt reactor with fast neutron spectrum. It is shown that the efficient transmutation of all existing transuranium isotopes would be possible from neutronic point of view in a time frame of about 60 years. For this task three reactors of a mostly new technology would have to be developed and a twofold life cycle consisting of a transmuter operation and a deep burn phase would be required. A basic insight for the optimization of the time duration of the deep burn phase is given. Further on, a detailed balance of different isotopic inventories is given to allow a deeper understanding of the processes during transmutation in the molten salt fast reactor. The effect of modeling and simulation is investigated based on three different modeling strategies and two different code versions.

## Introduction

The German government has decided to shut down eight nuclear power plants (NPPs) immediately, in April 2011. This was a consequence of the Fukushima event in March 2011. The decision has been approved by the change of the ‘Atomgesetz’ in August 2011. Further, it has been determined that the remaining 9 NPPs will be staggered shut down, with the last two ones in 2022 [1]. Several studies have been performed to estimate the transuranium isotope (TRU) accumulation due to the operation of the NPPs. The following approximate TRU amounts in Germany at the end of the nuclear reactor operation period have been reported in former studies: ~135 t plutonium, ~13.5 t americium, and ~1.7 t curium [2, 3, 4]. The estimations on the TRU amounts had to be corrected slightly due to the phase out decision and the rapid close down of the eight plants. The most recent studies considering the nuclear phase out predict now an estimate composition of 131 t plutonium, 6.2 t neptunium, 14.6 t americium, and 0.7 t curium [5] for 2022, when all nuclear plants are out of operation. These amounts of TRU, or their evolutionary results, are foreseen to be put to a final repository when/if a location is found. The complicated challenge to find a final disposal site is currently tackled by the politics with a new site selection law [6]. In addition to the final disposal of the heat producing waste in deep geologic formations, the waste management strategy of Partitioning and Transmutation (P&T) is under intensive discussion in Germany now [7, 8]. The partners in the German P&T study [7] defined the overall amount of TRUs to be transmuted to 170 t. The discussion about different waste management strategies in Germany is reflected in the site selection law. Following the law, a commission has been installed with the task to decide if other possibilities for the handling of the heat producing waste can be envisaged and consequently, the transfer into a final disposal should be postponed [6]. In general, it should always be kept in mind that P&T cannot replace a deep geologic disposal. Moreover, the transmutation of the TRUs will lead to an increase of the amount of fission products to be stored in the final disposal. However, most of these fission products have a significantly shorter half live than the TRUs. Consequently, P&T has the potential to reduce the long term challenges to be solved in the design of a deep geological disposal. The following chances have been identified in the German study on P&T [7]. The application of P&T on industrial level has the potential for:

- a significant volume reduction of the high level waste which has to be disposed. The main reason for the strong reduction is the separation of the actinides remaining after the unloading of the fuel from the nuclear reactor. All actinides will be removed from the waste stream. Finally, this leads to a significant reduction of the required final disposal space. Additionally, the separated uranium can be reused.
- a significant reduction of the long term activity of the materials in the high level waste disposal. The activity of the waste after the application of P&T and after 1000 years of storage time is comparable to the activity without P&T after one million years storage time.
- a plutonium content in the final disposal which is negligible after the application of P&T. Thus, there is no risk of misuse and theft of Plutonium anymore.
- the possibility of improved, selective conditioning of mobile fission product. This approach further reduces the risk of releases from the final disposal.
- a strong reduction of the heat production of the high level waste after an intermediate storage time of 70 years and more. Removing the Americium has the potential to reduce the required final disposal space due to possible denser pacing of the waste packages.

Two different scenarios for the application of P&T have been investigated and discussed within the study, an European and a national one. Two main options have been calculated for the national scenario: ADS with fertile-free fuel and FR with fuel containing fertile isotopes [7]. Molten salt reactors have been mentioned but not evaluated in detail within the study. To clarify the range of possible P&T options, the application of molten salt reactors will be investigated in this work. One interesting point of the discussions was the opportunities which are given by the molten salt reactor technology. This technology, originally developed in the 50ies and 60ies, has attracted some new interest which was focused in the EURATOM project MOST—review on MOltenSalt reactor Technology [9, 10]. Following the MOST project, the EVOL project has been launched [11, 12] and the Russian Project: “Minor Actinides Recycling in molten salts” (MARS) [13, 14, 15]. A three-year Euratom-Rosatom collaboration has been established, through the parallel coordinated projects (MARS-EVOL) on MSR [16]. The new interest in molten salts and the corresponding reactor technology can be explained by some really interesting features of the molten fluoride salts. Major points are the wide range of solubility of actinides, the thermodynamic stability, the resistance against radiologic decomposition, the low vapor pressure at operation temperature, and the compatibility with nickel based alloys as construction material [17]. In addition to the specific features of the fluoride salts, molten salt fast reactors (MSFRs) exhibit large negative temperature and strongly negative void reactivity effects which lead to a favorable stable operational behavior. Uri Gat et al. [18] proposed the MOLTEN SALT REACTORS FOR BURNING DISMANTLED WEAPONS FUEL based on their experience already in 1992. In their abstract they have summarized some really strong arguments which make molten salt reactors highly interesting for the burning of plutonium: “The MSRs have the flexibility to utilize any fissile fuel in continuous operation with no special modifications, as demonstrated in the Molten Salt Reactor Experiment, while maintaining their economy. The MSRs further require a minimum of special fuel preparation and can tolerate denaturing and dilution of the fuel. Fuel shipments can be arbitrarily small, which may reduce the risk of diversion. The MSRs have inherent safety features that make them acceptable and attractive. They can burn a fuel type completely and convert it to other fuels. The MSRs also have the potential for burning the actinides and delivering the waste in an optimal form, thus contributing to the solution of one of the major remaining problems for deployment of nuclear power” [18]. One of the most attractive features of liquid fuelled reactors is; when the plutonium is once put into a molten salt reactor system it is not necessary to handle or take out the plutonium outside of the reactor system in any of the steps of the burning operation anymore [18]. This is an advantage which is in strong contrast to the fuel management like it is to be applied for any kind of solid fuelled reactor with the separated steps of reprocessing, fuel production, and fuel irradiation including the transport between the sites. In a molten salt reactor neither external reprocessing, nor fuel management or transports are needed. In this kind of reactor only the fission products have to be separated out from the salt, which is typically done in an online process on the site. A presentation on the possible application of molten salt reactors for burning of plutonium and minor actinides has already given at the mentioned NATO workshop in Moscow. The authors highlighted the possible all in one solution and the flexibility of the system for the use of different fuels, too [19]. From the point of view of the nuclear fuel cycle, molten salt reactors have been a part of the U. S. study on Comprehensive fuel cycle options. In the study some really interesting and important advantages have been worked out in the comparison with different other kinds of reactors like the very low loss of fissile material in the processing and the extremely high fissile material utilisation [20].

**Fig 1:**
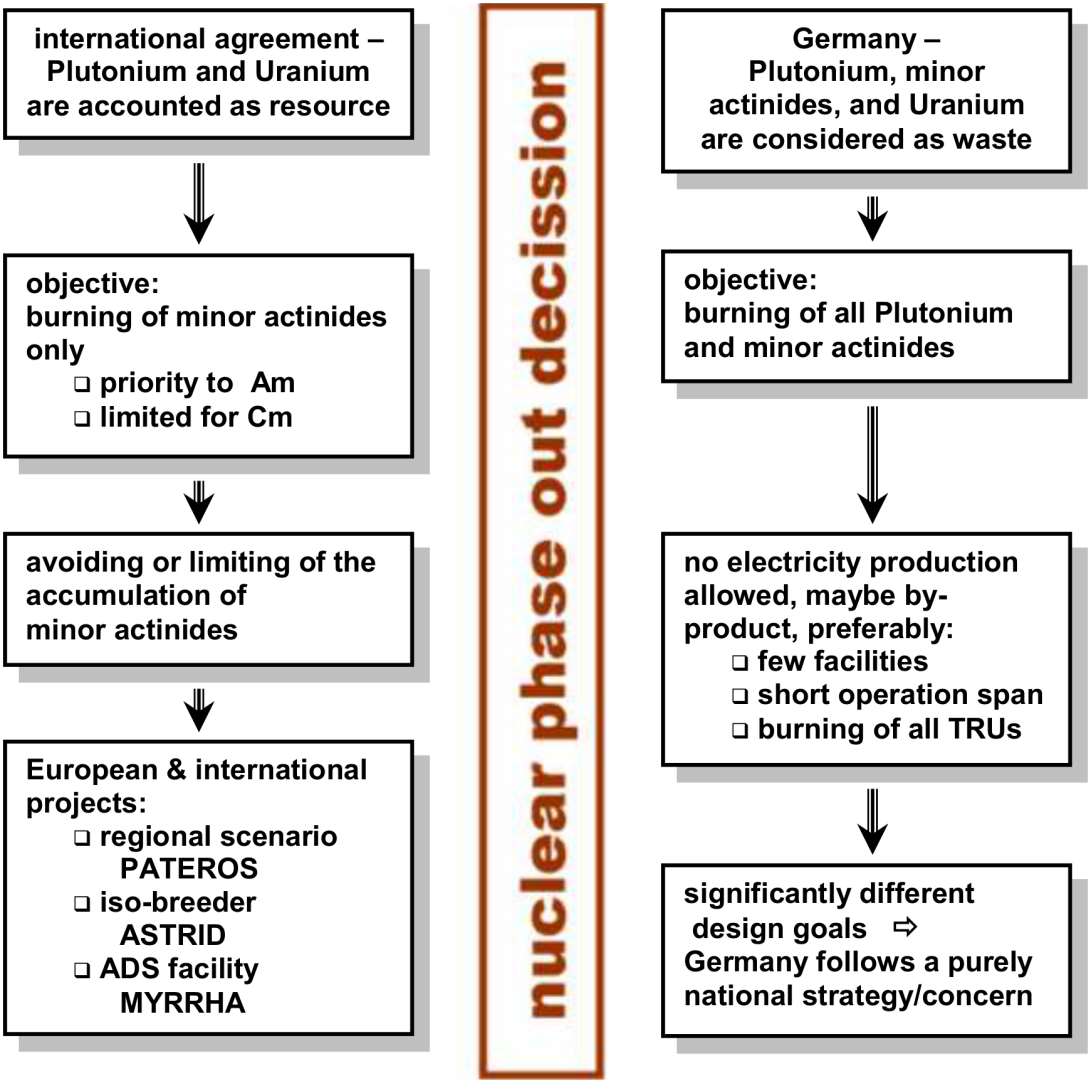
The specific situation for the application of P&T in Germany in comparison with the international view.

Several scenario studies have been performed in the last decades. Some of them even take the phase-out into consideration. The most prominent ones have been performed in EC-FP5 Red-Impact [21] and EC-FP6 PATEROS [22] under the roof of European framework programs. Additional important contributions have been given by the works of the OECD/NEA in the frame of the Working Party on Scientific Issues of the Fuel Cycle (WPFC)[23], and by the IAEA studies in the INTERNATIONAL PROJECT ON INNOVATIVE NUCLEAR REACTORS AND FUEL CYCLES (INPRO) [24]. However, the conditions of the phase out in Germany define some very special boundary conditions which have not been considered in these studies. The details are given in the schematic view in Fig 1, below. The key point is the perception of the worth of plutonium in Germany. It is no longer seen as a valuable resource it is accounted as waste which would have to be burnt in the case of transmutation. Thus the primary objective of the design of a transmutation facility has to be the efficient burning of the TRUs, the produced energy will only be a by-product and not the main product in contrast to classical fast reactor designs with fertile isotopes in the fuel.

As a general prerequisite for successful P&T, the partitioning technology for the already existing LWR fuel has to be developed to industrial standard, either in Germany or in co-operation within Europe. Transmutation of all TRU isotopes of the burnt LWR fuel faces an additional problem in the case of a nuclear phase out decision. It has been intensively discussed during the P&T study [7], that P&T is only an attractive and useful solution, if all TRUs can be burnt. In traditional transmutation schemes and the corresponding scenario studies which achieve high TRU burning rates, it has been always supposed that the reactor operation is continued. Thus, it is not necessary to care for the TRU amount which is loaded inside the reactor park. This TRU amount is required for the operation of the reactors. When the reactor operation is stopped at all, the situation is completely different. In this case the load of the last reactor plays a significant role. The last loading can accumulate to ~5% to 15% of the overall TRU amount to be burnt in Germany, depending on the transmutation strategy and the reactor design. Thus, it would make the implementation of the P&T process very questionable when this amount of TRU would have to be transferred into the final disposal. In this case there would be no gain in reducing the requests for a final disposal, since the long term activity and radiotoxicity would still be dominated by the TRUs. The described problem is known as the last transmuter problem. Recently, a solution for this problem has been proposed in a generic study based on the EVOL molten salt reactor configuration. The basic idea is the implementation of a twofold operation cycle [25]. The described reactor operation consists of a transmuter mode feeding the transuranium isotopes. It is followed by a deep burn phase. Here, the bred U-233 from the blanket is used as fissile material to burn the last TRU load very efficiently. In this phase, the reactor is operated on uranium as fissile material. This has the consequence of slight increase of the amount of fission products which has to be put into the final disposal. This surplus of fission products is caused by the slightly reduced overall TRU burning rate, see discussion of the results. At the end of the deep burn phase the remaining uranium could be forwarded to reactor operation in another country. This can be done without proliferation concerns as long as it is diluted with U-238 to the required fissile content defined in the non-proliferation guidelines. First publications on the application and the optimization of molten salt fast reactors with a specific focus on P&T and the special German situation have already been published as a consequence of the German P&T study [26, 27,28].

The calculations in this publication are based on the twofold operation cycle to answer the major questions defined by the special requests which are given by the German situation. The requests can be deduced from the major objectives found in the P&T study:

- as few facilities as possible
- as short operation span as possible
- burning of all TRUs without a leftover of TRUs

This leads to the research question:

Is transmutation under the objectives of the nuclear phase out decision feasible from neutronic point of view?

Here, the major points are: how many transmutation reactors would have to be operated for how long? Will it be possible to burn more than 99% of the TRUs besides possible losses in the salt clean up system? The simulated reactor configuration including the developed model of the reactor and the applied code are described in detail in the following materials and methods section. This section is followed by the section results which includes the achieved results as well as the discussion. It is complemented with an analysis to gain a deeper insight into the transmutation processes going on in the simulated reactor during the life time.

## Materials & Methods

### Data Availability

The raw data of the calculations and the used PYTHON script are stored under https://www.hzdr.de/db/!FzrTools.Archiving.ArchiveInfos?pNid=223&pId=1548 Due to international standards on nuclear safety and security, the data is available from the HZDR archive upon request. Please contact the HZDR library (library@hzdr.de) to request the data.

### Reference Configuration

**Fig 2:**
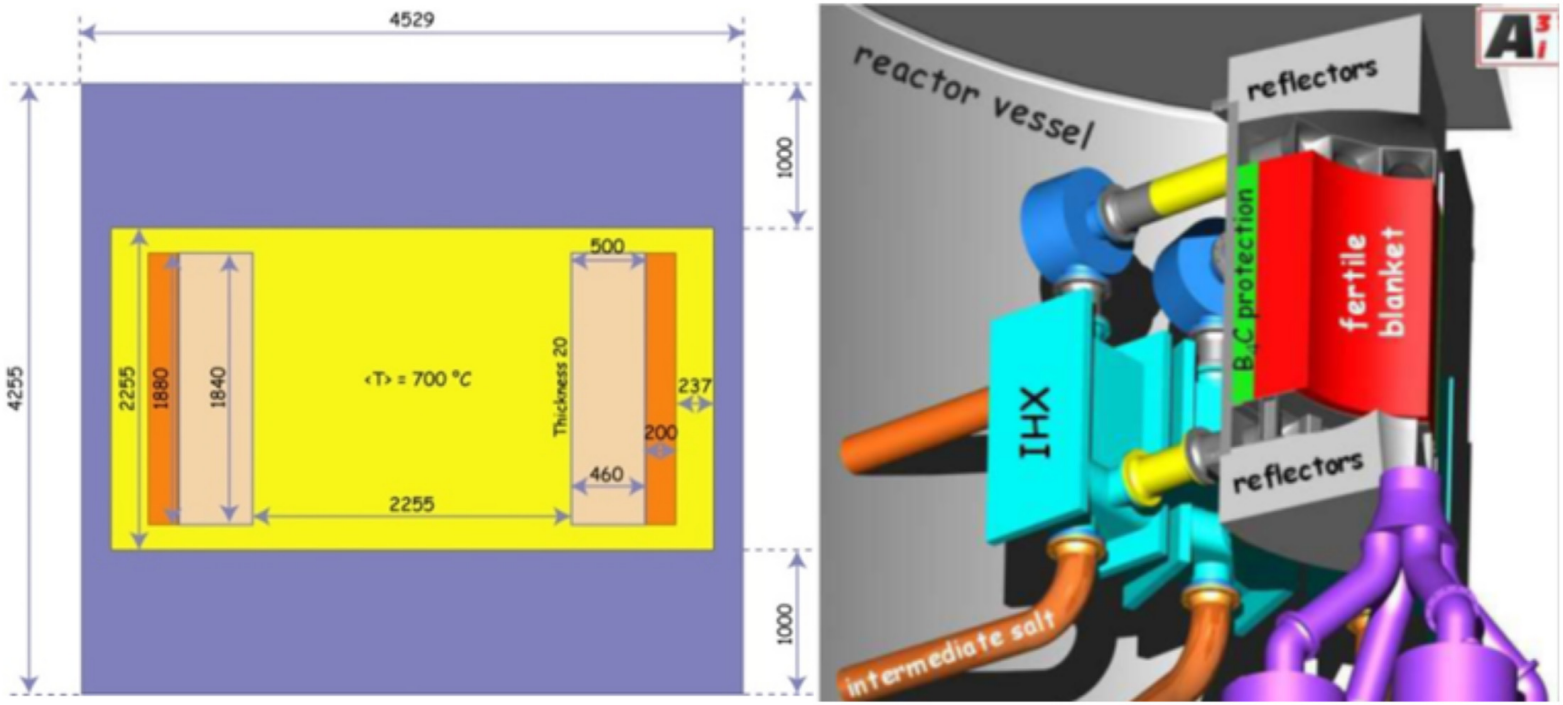
(Right): Simplified scheme of the MSFR system including the core, blanket and heat exchangers (IHX)–(Left): Benchmark definition [30].

The calculations are based on the core dimensions and the boundary conditions given in the EVOL benchmark definition (see Fig 2). The reference is a MSFR with 3000 MW_th_ and a fast neutron spectrum. In a first approach, which has been refined during the project, the core is a single cylinder. The nuclear reactions occur within the flowing fuel salt inside the cylinder [29]. The dimensions are shown in Fig 2. The core is composed of four volumes: the active core, the upper extraction area, the lower injection area, and the out of core area with the heat exchanger and the pumps. The used TRU isotopic vector (see Table 1) follows the definition of the EVOL benchmark. It is given by the configuration to be expected after a single use of UOX in a PWR to a final burnup of 60 GWd/tHM after five years of storage [29]. The TRU isotopic vector is one generic configuration. In the case of a real transmutation, the TRU isotopic vector would vary since the TRUs will not be pooled after reprocessing due to criticality reasons. Additionally, the TRU vector undergoes some changes due to the decay of some TRU isotopes, mostly Pu-241 and Cm-244. Generally, the TRU isotopic vector depends:

- strongly on the burnup of the fuel
- strongly on the fuel type, UOX fuel or MOX fuel
- strongly on the storage time, due to the decay of Pu-241 to Am-241
- weakly on the reactor type, PWR or BWR

**Table 1:** Used TRU isotopic vector—EVOL benchmark data.

Np 237	6.30%
Pu 238	2.70%
Pu 239	45.90%
Pu 240	21.50%
Pu 241	10.70%
Pu 242	6.70%
Am 241	3.40%
Am 243	1.90%
Cm 244	0.80%
Cm 245	0.10%

During the real reactor operation the amount of refill of TRUs has to be adapted to the isotopic vector. This is an additional parameter which will vary the requested refill like the criticality level of the core. The use of only one TRU isotopic vector seems to be adequate for the approximation level required for this kind of long term study for the proof of the feasibility.

### Modeling and Calculation Tool

**Fig 3:**
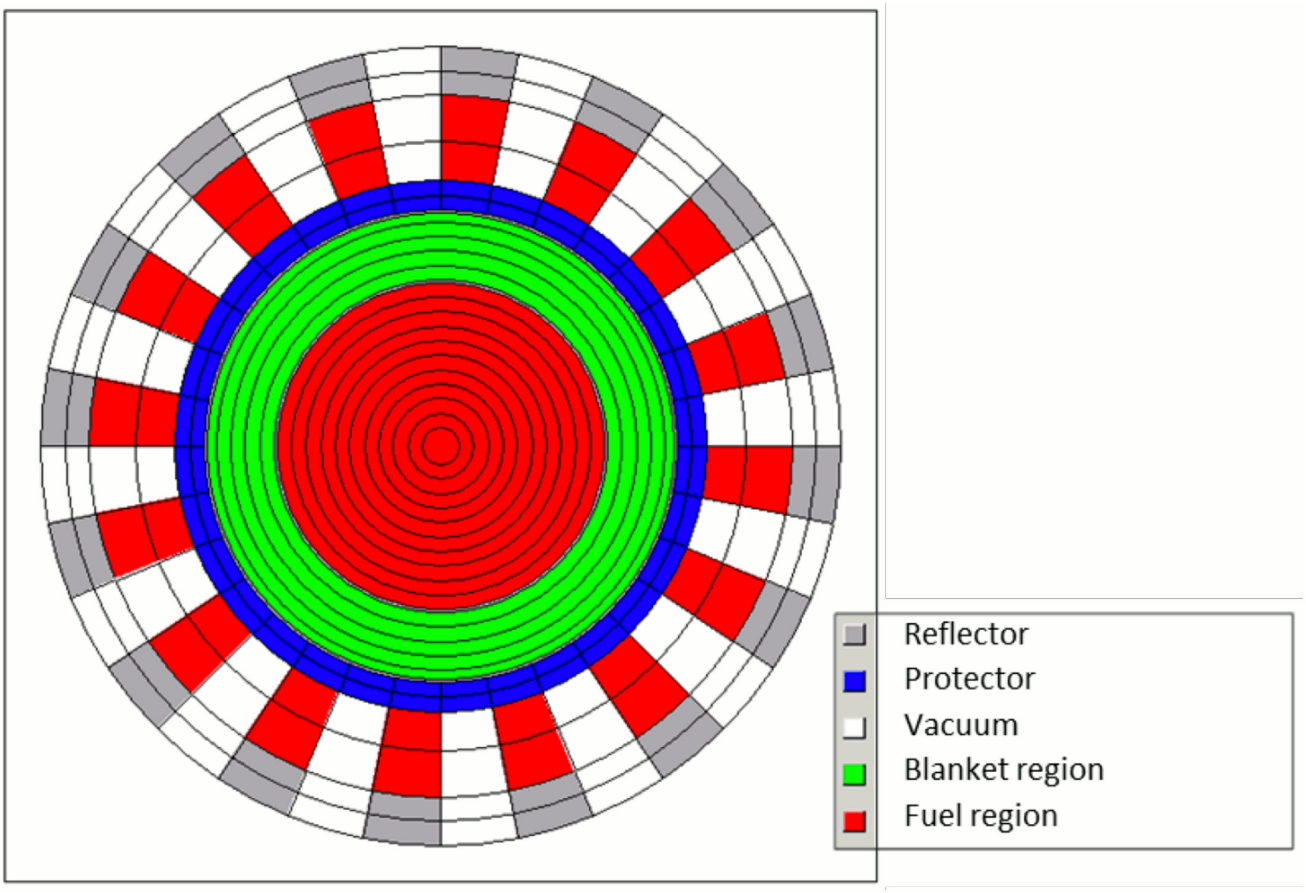
Volume corrected 2D HELIOS model of the molten salt reactor.

For the simulations, the HELIOS code system is used in two different versions: HELIOS 2.1 with the internal 49 group library [31] and HELIOS 1.10 with the internal 47 energy group library [32]. The choice for the number of energy groups is a compromise between the level of approximation and the requested calculation time. However, it seems to be adequate for the current status of the study. In both versions, the code is a 2D spectral code with wide unstructured mesh capabilities and a transport solver, based on the collision probability method [33]. The benchmark configuration is transferred to a volume corrected 2D HELIOS model (see Fig 3). The model has been adopted for an as close as possible reproduction of the 3D structure and the relations between the different materials to care for the 3D effects like the reduced height of the blanket compared to the core in the benchmark description This correction leads e. g. to a reduced thickness of the blanket in the adopted 2D model. The leakage in the third dimension is incorporated by the insertion of a buckling correction available in HELIOS (BSQ: 0.00002). The value for the correction has been fixed by a comparison of 2D and 3D calculations within the EVOL benchmark exercises. With this setting k of ~1.005 is required for a pseudo 3D k_eff_ of 1.0. The leakage in radial direction is directly modeled. Additionally, the model has been detailed compared to the EVOL benchmark configuration. The 16 heat exchanger pipes are resolved for a better representation of the real geometry.

**Fig 4:**
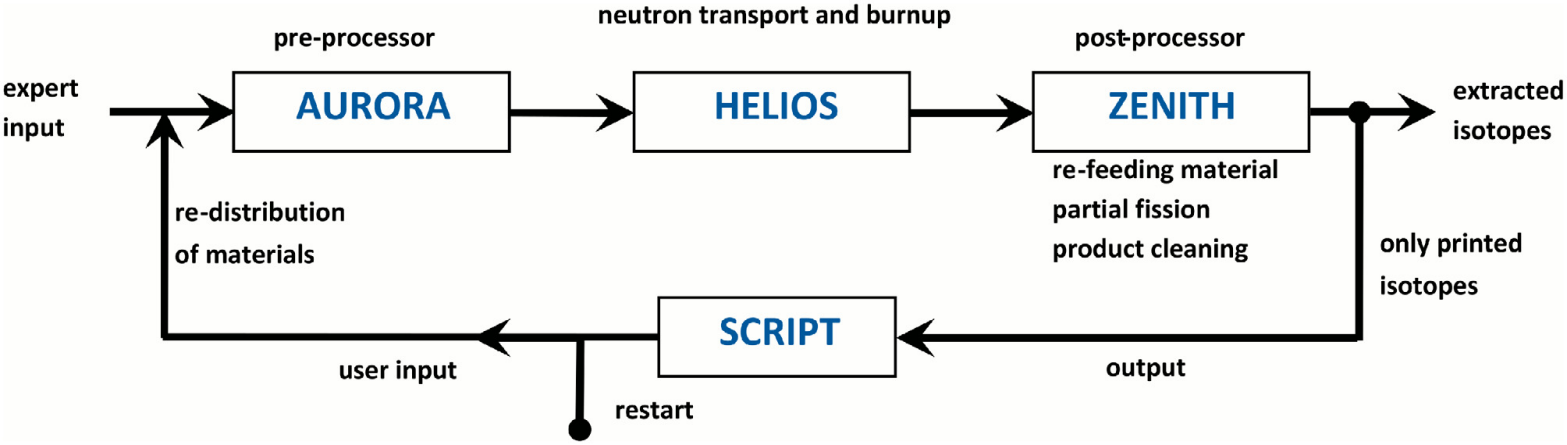
Description of the calculation cycle for the simulation of a MSR.

The HELIOS code is an industrial standard software which performs the neutron transport calculation, the burn up calculation, and if requested the cross section preparation in defined calculation areas. Originally, the HELIOS code has been written for the simulation of solid structured fuel assemblies. The possibility of online refueling and online reprocessing is not foreseen. To deal with these special features of molten salt reactors a PYTHON script has been developed, which is based on the special features of the HELIOS package. All input data, which is constant during the whole reactor operation, is stored in a so-called expert input. The changing material configuration is given in the user input which is written new in every cycle using the PYTHON script. Within each cycle 5 burnup steps are calculated in each HELIOS run. Both inputs are merged in the pre-processor AURORA, which creates the updated input for the HELIOS run for the determination of the neutron flux distribution and the burnup of the materials. The results are finally evaluated for each cycle in the post-processor ZENITH. Here it is decided which isotopes will be fed back into the next user input which is created with the help of the PYTHON script (see Fig 4). Theoretically, it is possible to simulate a molten salt reactor precisely by using small time steps in this calculation loop. In a real MSR two different time scales for the salt cleanup can be observed, due to the different extraction methods for the fission products. There are the helium bubbling for the gaseous and the volatile fission products with a comparably short acting time and the online salt cleanup for the dissolved fission products with a significantly longer acting time. A new strategy has been developed. It is characterized by the use of a reduced burnup per cycle (5 GWd/tHM using five burnup steps in HELIOS). It is coinciding with a full removal of the gaseous and the volatile fission products. At the end of the cycle, only a partial removal of the dissolved fission products are removed (16.6%, and 15% for the lanthanides). This methodology leads to a full clean up time of ~450 days, as it has been defined in the EVOL benchmark description. The recovery of the U-233 and Pa-233 from the fertile salt in the blanket will be performed in a real MSFR with a process which is very comparable to the salt cleanup process. The speed of processing has to be determined in the design of the blanket size and configuration. The process is modeled by withdrawing both materials at the end of each cycle from the isotopic configuration of the blanket salt. The amount of U-233 which can be harvested from the blanket depends strongly on the dimension and the composition of the blanket. This optimization has to be performed in conjunction with the decision how long the deep burn phase should last. However, it has to be accepted that there is a physical limit for the amount of U-233 which is determined by the available amount of neutrons for the breeding process.

The fuel salt configuration differs significantly from the EVOL benchmark definition since a fertile free configuration is requested for the simulation. This configuration would not be possible using the EVOL salt configuration due to the characteristic solubility limits for the TRUs. Here, one of the salt configurations and the corresponding thermodynamic data of the MOSART project [14] is used, 17LiF-58NaF-25BeF_2_ (mole%) mixed with TRUF_3_. This salt composition has been developed for the use in a fertile free system. The salt composition in the fertile region is kept from the EVOL benchmark (LiF-ThF_4_).

Due to the characteristics of HELIOS, some approximations have to be accepted. There is no fuel salt movement, thus an undesired burnup distribution arises during the cycle. The materials are only re-distributed when a new user input is defined. This approximation will be improved due to the halving of the burnup per cycle and the partial removal of the fission products. HELIOS is a LWR code. It is mostly validated for the UOX and MOX fuel, but on a lower extent for the Thorium fuel cycle, too. A LWR spectrum is used for the weighting of the master libraries inside each energy group. However, comparisons to SERPENT on the isotope accumulation during the burnup in a fast reactor configuration using different HELIOS libraries have shown an acceptable agreement for the major isotopes [34]. Additionally, different versions of the HELIOS 1 code have been used in the EVOL benchmark calculations for MSFR [35] and in the benchmark calculations in the ESNII+ project for SFR [36]. In both benchmark calculations the obtained benchmark results were on a comparable level to the other applied codes. No major systematic discrepancies have been observed. A significant improvement has been observed for the use of HELIOS 2 with the cross section set based on ENDF/B VII. The approximations and the use of the HELIOS code package seem to be adequate for the approximation level required for this kind of long term feasibility study. The major uncertainties are given by the current very preliminary design. It is far away from any detailed design which would be requested for an elaborate study. These design uncertainties are expected to cause significantly higher uncertainties to the results than the approximations in the modeling and the applied code. In general, the calculations are performed to demonstrate the feasibility of burning TRUs efficiently under the very restrictive boundary conditions of the phase out decision.

An overview on the main assumptions, the major modelling parameters, and the verification of HELIOS 2 is given in Table 2.

**Table 2:** Main assumptions in code, modelling, and comparison to SERPENT.

Number of energy groups	177 groups weighted with LWR spectrum within group
Length of calculation cycle	5000 MWd/tHM
Actinide isotopes followed	U-232, U-233, U-234, U-235, U-236, U-237, U-238
	Np-237, Np-238
	Pu-238, Pu-239, Pu-240, Pu-241, Pu-242
	Am-241, Am-242m, Am-243
	Cm-242, Cm-243, Cm-244, Cm-245, Cm-246, Cm-247, Cm-248
	Bk-249
	Cf-249, Cf-250, Cf-251, Cf-252
Salt clean-up simulation	all volatile fission products
removal at the end of each	16.6% of the dissolved fission products
5000 MWd/tHM cycle	15% the lanthanides
2D to 3D corrections	radial leakage treated in code
	axial leakage adjusted
	volume adjusted
Difference HELIOS 2 to	U-235 <0.5%, U-238<0.5%
SERPENT for compared isotopes up to 90GWd/tHM	Pu-238<0.8%, Pu-239<2.0%, Pu-240<0.25%, Pu-241<0.2%
taken from [34]	Am-241<0.5%, Am-242m<1.0%, Am-243<1.5%
	Cm-242<1.25%, Cm-243<1.25%, Cm-244<2.5%

## Results

### Simulation over the Lifetime

The simulation results are achieved by using the script, described above, and the HELIOS code. The operation time in the transmuter mode is determined by the expected number of reactors and the amount of TRU in Germany (170 tons) which has to be burnt in these reactors. A first analysis based on HELIOS 1 calculations and the use of the reduced models has indicated that three reactors are the most attractive configuration [37,38]. The transmuter mode is followed by the deep burn phase, where no TRUs are inserted into the reactor anymore [25]. The requested fissile content is in this phase established by the feeding of U-233, which has been bred in the LiF-ThF_4_ salt in the fertile blanket. This blanket is not only required for breeding, it is also an important structure for the shielding of all components, the outer vessel and the major parts of the primary circuit, the heat exchangers as well as the pumps. The size of the blanket has to be optimized to achieve an optimal shielding [39,26]. The salt composition and the uranium extracting system have to be designed based on the anticipated operation time of the reactor in the deep burn mode.

**Fig 5:**
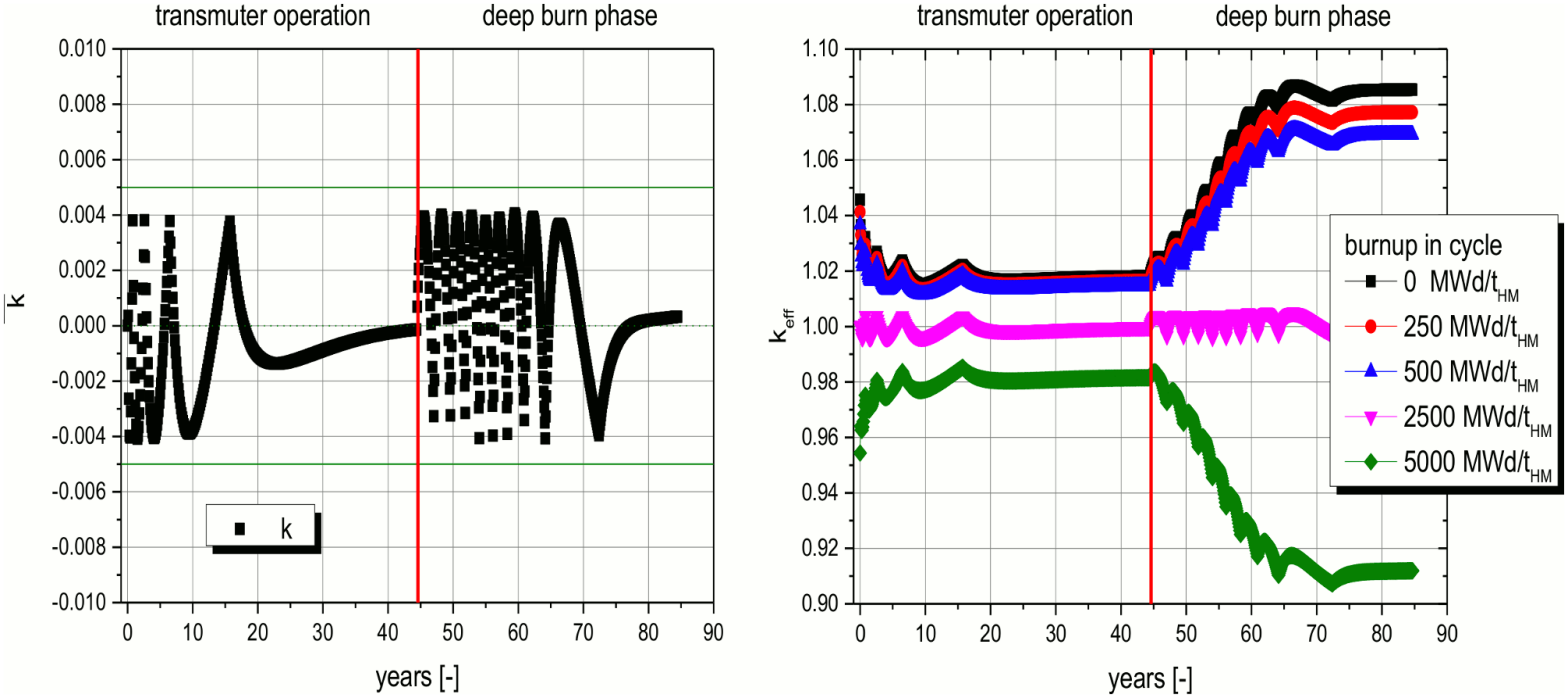
Cycle averaged k deviation (left) and burnup dependent k_eff_ values in each cycle (right)over the whole operation time.

**Fig 6:**
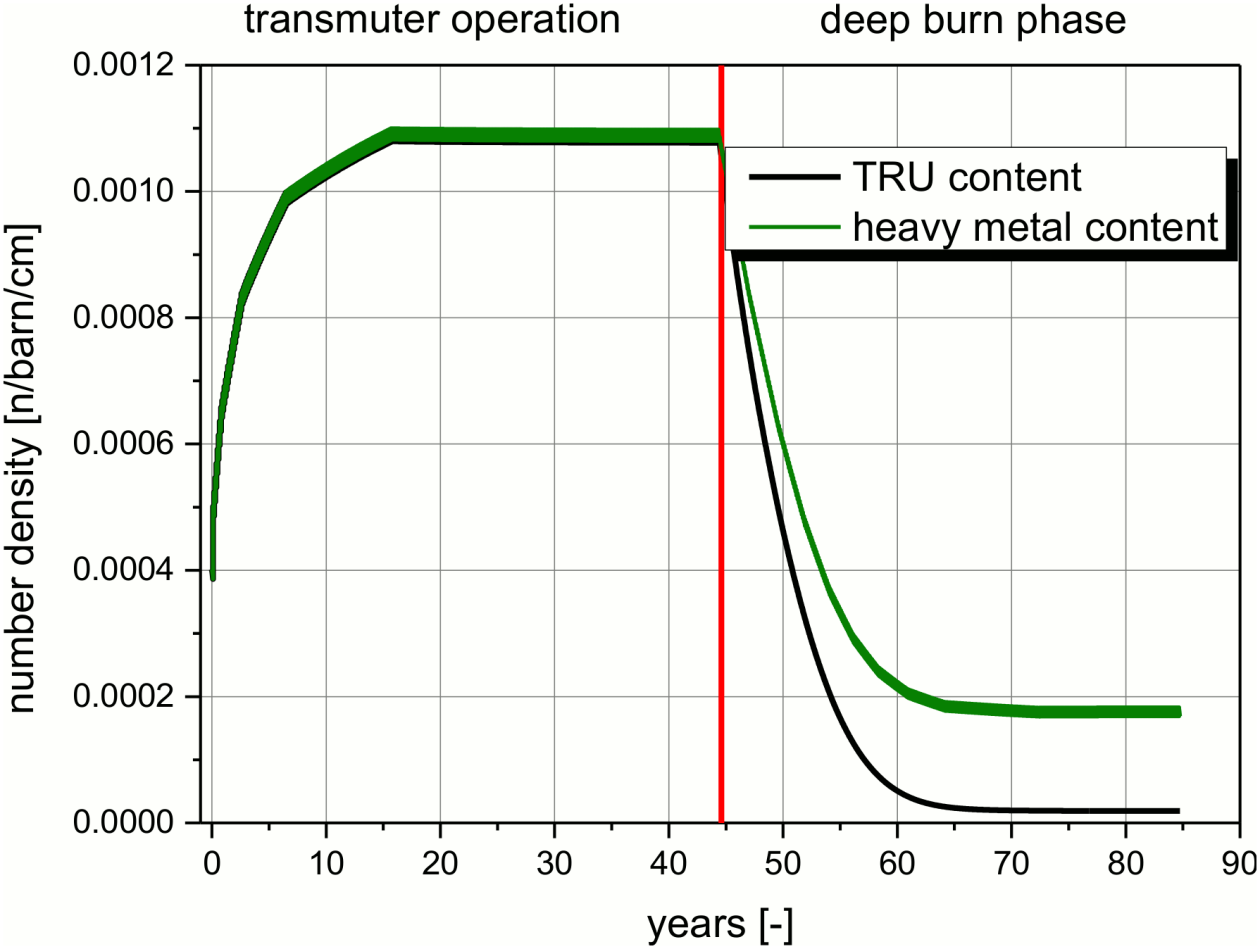
Change in the integral heavy metal and the TRU content in the core over the operation time.

The multiplication factor in the calculation of the cycles is controlled during transmuter operation as well as in the deep burn phase. This task is performed via the feeding of fissile material which has to be adopted during the life of the reactor several times to keep the cycle averaged multiplication factor always in a band of ± 0.005 around criticality. This process of continuous insertion of fissile material to compensate the burnup of fissile material will take place in the real reactor, too. However, the process will be performed in a more continuous way than in the simulation. This kind of process is typical for all kinds of reactors with the possibility of continuous feeding, like molten fuel reactors or pebble bed high temperature reactors. The curve for the deviation of the averaged multiplication factor swings in the given band of ± 0.005 around criticality during the whole observation period of more than 80 years (see Fig 5, left). The Δk curve stabilizes in the transmuter operation after around20 years. This is a consequence of the increasing amount of TRUs in the core (see Fig 6) during the transmuter phase. The increasing amount of even plutonium isotopes (seelater dicussion) plays the major role. These isotopes act as fertile material for the breeding of new fissile material. The mostly fertile isotopes accumulate to an asymptotic amount in the first 10 to 20 years of operation. This process leads to the significantly increased TRU content in the core, compared to the starting value and causes the observed stabilization. The effect can be observed in the averaged Δk curve (Fig 5, left) as well as in the burnup dependent k_eff_ values in each cycle (Fig 5, right).

The TRU content and as a consequence the content of heavy materials in the reactor core reduces during the deep burn phase significantly. Thus the stabilizing amount of fertile material decreases rapidly. This process leads to a system which is much more sensitive to the addition of the fresh fissile material at the beginning of each cycle. This is reflected in the much wider spread and the quicker changes in the averaged Δk values. The significantly steeper burnup curve in the cycles is reflected by a wider spread of the burnup dependent k_eff_ values inside the cycles (Fig 5, right). The real end of operation has to be determined by the requested burnout of the TRUs.

Compared to the starting value, the TRU content increases by nearly a factor of three during the transmuter operation (see Fig 6). The asymptotic amount of the TRU content approaches during the calculated transmuter operation the solubility limit for TRUs in the carrier salt with 3.01 mole %. This characteristic value for the salt has been determined experimentally to 3.0 mole % at 600°C. Using the formula given in [14] for the determination leads to 3.08 mole %. Thus the required carrying capacity offered by the salt, proposed in the MOSART concept using the EVOL configuration, is barely sufficient, within the calculation accuracy. Consequently, it is indicated that the temperature range proposed in MOSART (600°C inlet and 715°C outlet) should be increased slightly, maybe to the values proposed in EVOL (625 to 775°C) [30]. Additionally, this finding leads to the conclusion that the parameters for the optimization of the salt configuration should not only be the melting point of the salt and the demands for reprocessing. The observation of the carrying capacity for TRU isotopes is also an important parameter when a molten salt reactor is in discussion for transmutation purposes.

The heavy metal content in the core is equal to the TRU content during transmuter operation. A difference appears only in the deep burn phase, when U-233 is fed into the system as fissile material (see Fig 6). The most impressive fact is that the heavy metal content at the end of operation is less than the half of the starting value and less than 25% of the amount at the end of the transmuter operation. The explanation for this interesting fact can be found in the excellent fissile properties of the almost clean U-233 compared to the inserted TRU vector. This fissile material has been bred in the fertile blanket and is fed into the core. These properties have to be compared to the TRU vector with only ~55% odd Pu isotopes as excellent fissile material and 45% of isotopes which have a low fission cross section, even in a fast neutron spectrum.

**Fig 7:**
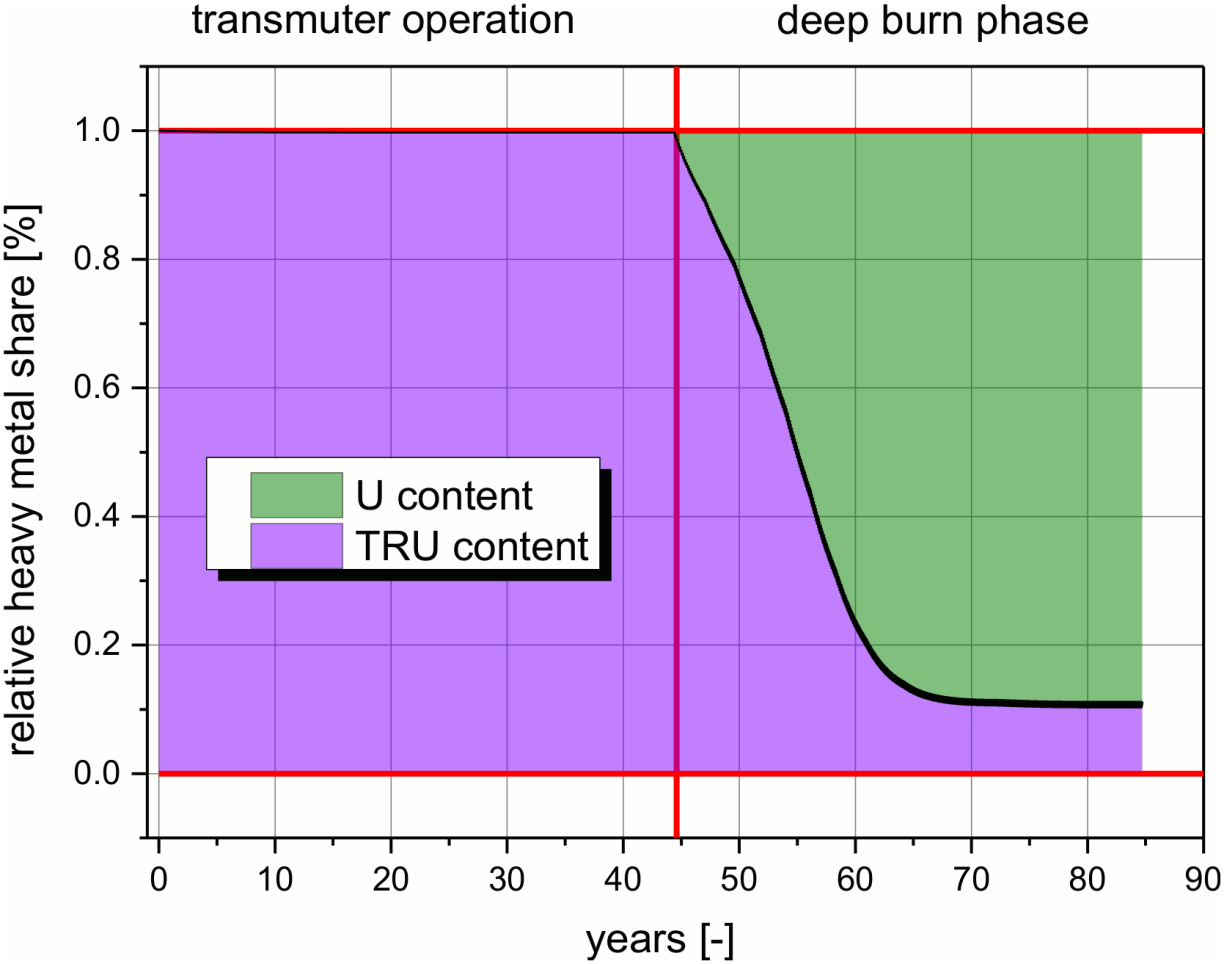
Relative share of fissile material in the core over the operation time.

The transition of the composition of the fissile share in the salt inside the core during the deep burn phase takes place in a continuous way (see Fig 7). This figure will be used to explain in detail the way of operation of the molten salt reactor and the transition from the TRU based reactor (transmuter operation) to the U-233 based reactor (at the end of the deep burn phase). In the transmuter operation, the reactor is all the time operated with pure TRU as fissile material which is fed into the reactor continuously. The integral amount of TRU material in the core rises during operation to an asymptotic value (see Fig 6), since the quality of the fissile material decreases due to absorption processes. More heavy TRUs are built up in breeding processes until an asymptotic value is achieved. With begin of the deep burn operation the feed is changed. The process is not an instantaneous switch of the reactor configuration, but a slow transformation. This is possible due to the continuous feeding process. The feed is simply switched from TRUF_3_ to ^233^UF_4_. All other components of the salt are not touched and stay in the reactor. The heavy metal configuration changes slowly over ~20 years of operation from the TRU dominated core at the start of the deep burn phase to the U-233 dominated at the end. Additionally, the integral heavy metal content decreases significantly in the deep burn phase (see Fig 6). The process of changing the fissile component which is fed into the system has already been applied in the MSRE [13]. From safety point of view or better from authority point of view, the change of the fissile material is expected to require a special operational permission. This task should be comparable to the operational permission which has to be received for a new loading of a LWR, when MOX fuel assemblies are foreseen to be inserted. Therefore, some additional safety analysis could be required. The U-233 is bred in the blanket during the whole operation time. In the EVOL proposal, the Uranium is foreseen to be continuously removed from the blanket. Thus it has to be stored, partly for a long time period. On the one hand, this storage has sure to be seen ‘critical’ in view of a possible proliferation risk. At the end of the transmuter operation ~8–10 t of U (U-233 with a small share of U-232, U-234, and U-235) have to be stored. On the other hand, in UK are currently ~75 t separated Pu stored (http://www.fissilematerials.org/blog/img/blog_globalPU_Oct2007_large.jpg) and the availability of separated fissile material is a problem appearing in every reprocessing facility. Thus, the methods for handling this kind of problem are already developed and demonstrated. However, this fact is a challenge for the safeguarding of the International Atomic Energy Agency. It will increase when any kind of P&T will be implemented in the future.

**Fig 8:**
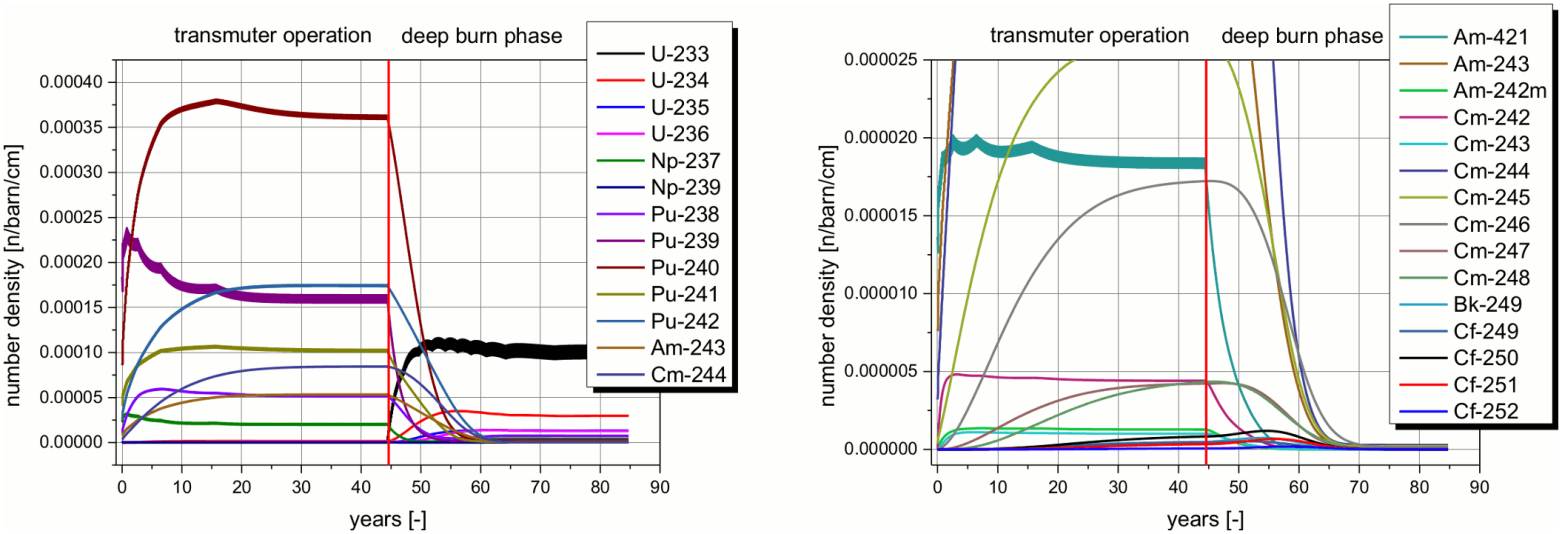
Change in the heavy metal isotope contents in the core over the operation time.

The detailed analysis of the isotopic composition of the heavy metal content in the reactor core (see Fig 6) during the whole operation is given in Fig 8. Almost all TRUs are accumulated during the transmuter operation. The slight changes in the feeding of the TRUs can be observed by the slow oscillations of the Pu-239 content, and on a lower extent all other isotopes which are fed. Additionally, there are very short oscillations visible for all isotopes which are fed into the system (PU, Am, and Cm), which can even not be resolved in the figure (increased thickness of the lines). These oscillations are created by the feeding and burning of the TRUs in each calculation cycle. The only exceptions are Pu-239, Np-237, and partly Am-241. The content of these isotopes decreases even though additional amounts of the isotopes are fed into the core at the beginning of each cycle. In the following deep burn phase, all TRUs are burnt very efficiently. First the Pu isotopes, followed by the Am isotopes, and finally by the Cm and the higher isotopes. However, this should not lead to the conclusion that the transmutation is not efficient during the transmuter operation. The observed accumulation during the transmuter operation is only an indicator that not all TRUs which are fed into the system at the beginning of each time step are burnt in the time period of the cycle.

In the deep burn phase, the uranium isotopes are accumulated (see black curve in Fig 8, left), but the main contribution is always U-233, only small amounts of even uranium isotopes are created. All TRU isotopes are burnt very efficiently during the deep burn phase, but at strongly different burning rates. First, the very good fissile materials and the low weighted isotopes disappear due to fission and capture processes. Especially, the higher curium isotopes like Cm-246 to 248 stay constant, or even grow slightly in the first five years of the deep burn phase. However, after about 5 years, these isotopes are burnt efficiently, too. A comparable behavior can be found for the very heavy curium isotopes. The amount grows in the beginning of the deep burn phase due to breeding processes from lighter isotopes. Finally, even the californium isotopes are burnt, since the built up of new californium isotopes decreases due to the reduced breeding potential. This is caused by the burning of the lighter precursor isotopes which could act as precursors for breeding.

An overview of the main operational results separated for the transmuter operation and for different options of the deep burn phase is given in Table 3. The most remarkable numbers are: in each of the requested three reactors 56.7 tons of TRUs are burnt. This amount is inserted over an operational period of nearly 45 years (transmuter operation). The TRU burning rate in the transmuter operation is already more than 85% of the inserted TRU. The remaining loading at the end of the transmuter operation is burnt in the deep burn phase. The time period for the deep burn phase depends on the requested burnout of the TRU isotopes. Thus, three reactors have to be operated for a bit more than 60 years to get rid of the overall 170 tons of TRUs to more than 99%. Any extension of the deep burn phase leads to counteracting consequences. On the one hand, the reduction of the TRUs improves up to 99.8%. On the other hand, the TRU burning rate decreases. This is caused by the extension of the deep burn phase. It leads to a shift of the energy production from the TRUs to the U-233. However, it has to be kept in mind that this very small remaining TRU amounts have to be put to the deep geological final disposal. Thus, the decision on the length of the deep burn phase is an optimization problem with boundary conditions which have finally to be defined by the public.

**Table 3:** Operational results for transmuter operation and deep burn phase.

operational results for transmuter		
thermal power	3000	MW
available TRU	170	tons
real TRU inserted (per reactor)	56.7	tons
operation time—transmuter	44.6	years
TRU burning rate (calc.)	42.4	kg/TWh
theoretical burning rate	42.3	kg/TWh
TRU burnt—transmuter*	86.4	%
deep burn 100 cycles		
overall operation time—transmuter + deep burn	54.5	years
overall TRU burning rate (calc.)	39.2	kg/TWh
TRU burnt—transmuter + deep burn*	97.5	%
deep burn 200 cycles		
overall operation time—transmuter + deep burn	62.9	years
overall TRU burning rate (calc.)	34.6	kg/TWh
TRU burnt—transmuter + deep burn*	99.6	%
deep burn 300 cycles		
overall operation time—transmuter + deep burn	71.2	years
overall TRU burning rate (calc.)	30.7	kg/TWh
TRU burnt—transmuter + deep burn*	99.8	%
deep burn 400 cycles		
overall operation time—transmuter + deep burn	79.4	years
overall TRU burning rate (calc.)	27.5	kg/TWh
TRU burnt—transmuter + deep burn*	99.8	%

* aside from possible losses in the salt cleanup system

The theoretically determined burning rate based on the energy per fission of plutonium and the burning rate determined in a detailed calculation of the transmuter operation over almost 400 calculation cycles (~45 years) agree very well. This result confirms the quality of the calculation procedure. In first order approximation, the amount of energy set free by the fission of the TRUs during transmuter operation will be the same for each fertile free reactor configuration since it is determined by physical constants. Thus, it is only the question if the fertile free configuration will be acceptable from safety, technological, and operational point of view. These requests are fulfilled in the MSFR due to the very special safety characteristics and the much simpler fuel production compared to solid fuels.

The uranium vector indicates a uranium composition with a fissile content of ~70% to ~80% and an amount of roughly 500 kg of fissile material for all stages of the deep burn phase longer than ~10 years. The detailed composition at the very end of the deep burn phase, after 400 cycles is given in Table 4.

**Table 4:** Uranium vector at the very end of the deep burn phase.

U-232	0.00023%
U-233	62,5%
U-234	19,8%
U-235	8,9%
U-236	8,5%
U-237	0,1%
U-238	0,3%

At any end of the deep burn phase a decision has to be made how to proceed with the remaining material with a fissile content of ~70 to 80%. If the material should be given to another country as fissile material, the uranium has to be blended in depleted uranium until the proliferation limit of 12% for U-233 and 20% for U-235 is reached. This process is already well established for the blending of weapon grade Uranium [40]. Finally, a general remark concerning radiotoxicity: the radiotoxicity content in the reactor is significantly reduced in the deep burn phase since the major carriers of the radiotoxicity are the TRUs.

### Material Balance Regarding Burning and Breeding

**Fig 9:**
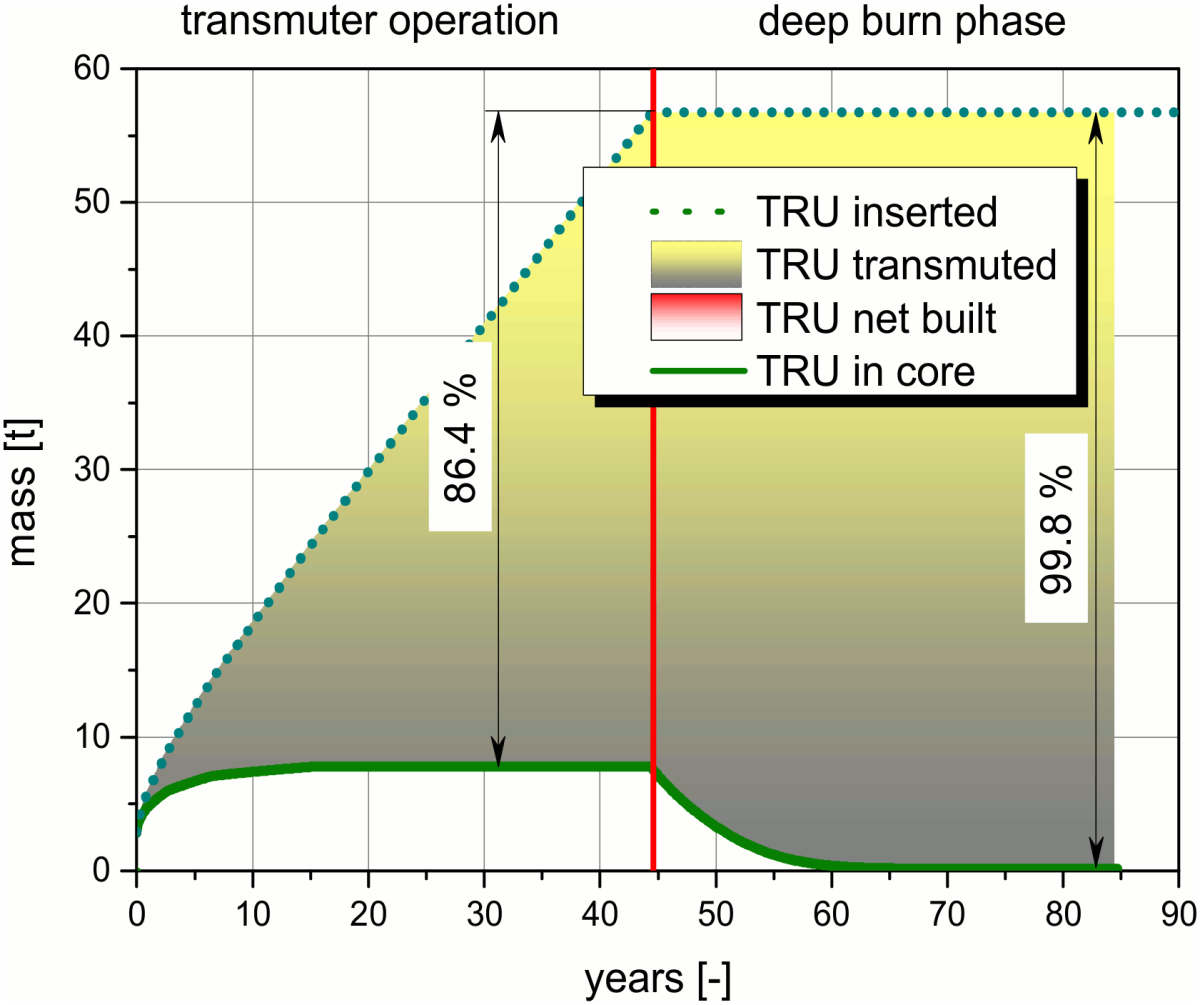
Comparison of the, into the core inserted, TRU mass and the, in the core resident, TRU mass over the operation time.

**Fig 10:**
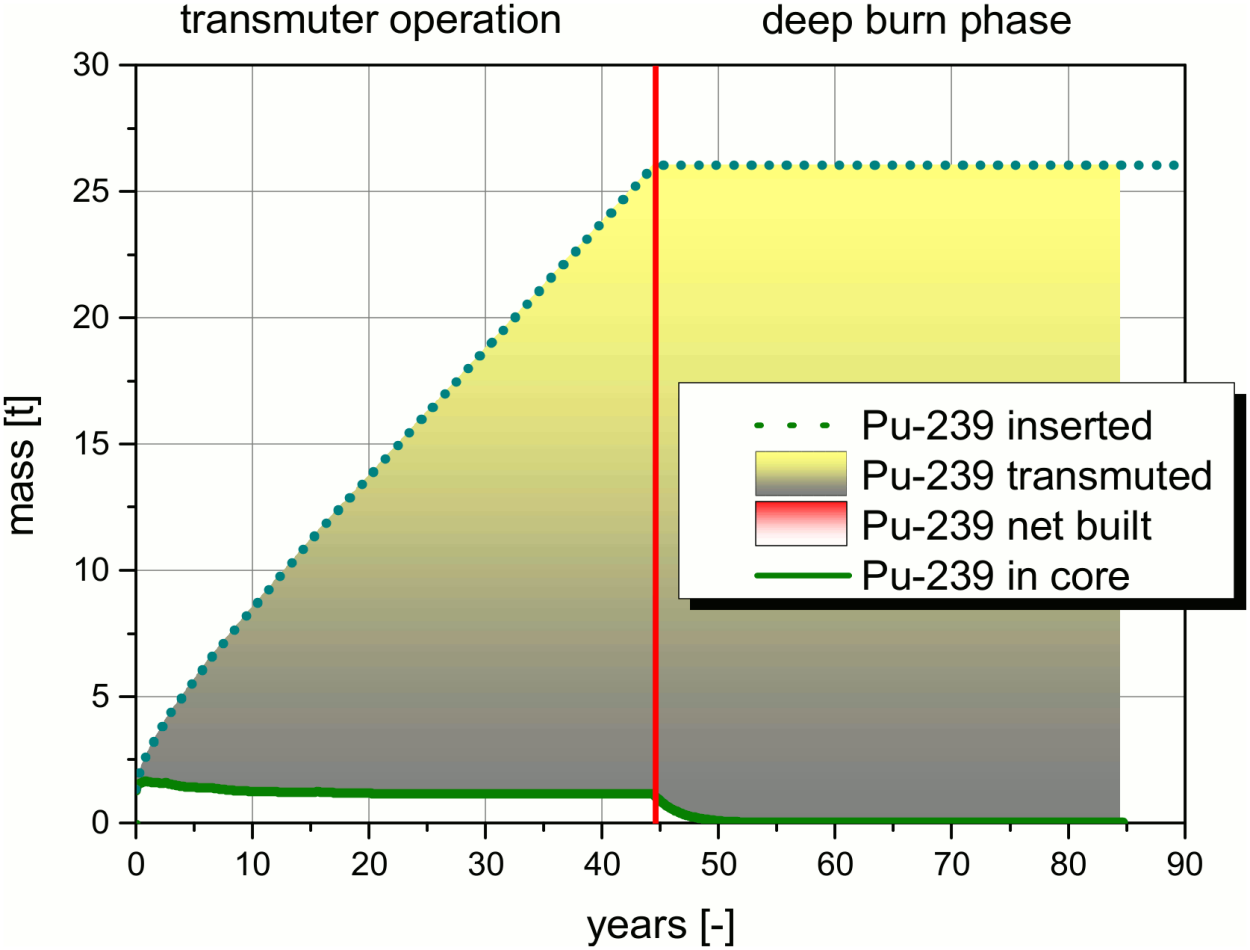
Comparison of the, into the core inserted, Pu-239 mass and the, in the core resident, Pu-239 mass over the operation time.

**Fig 11:**
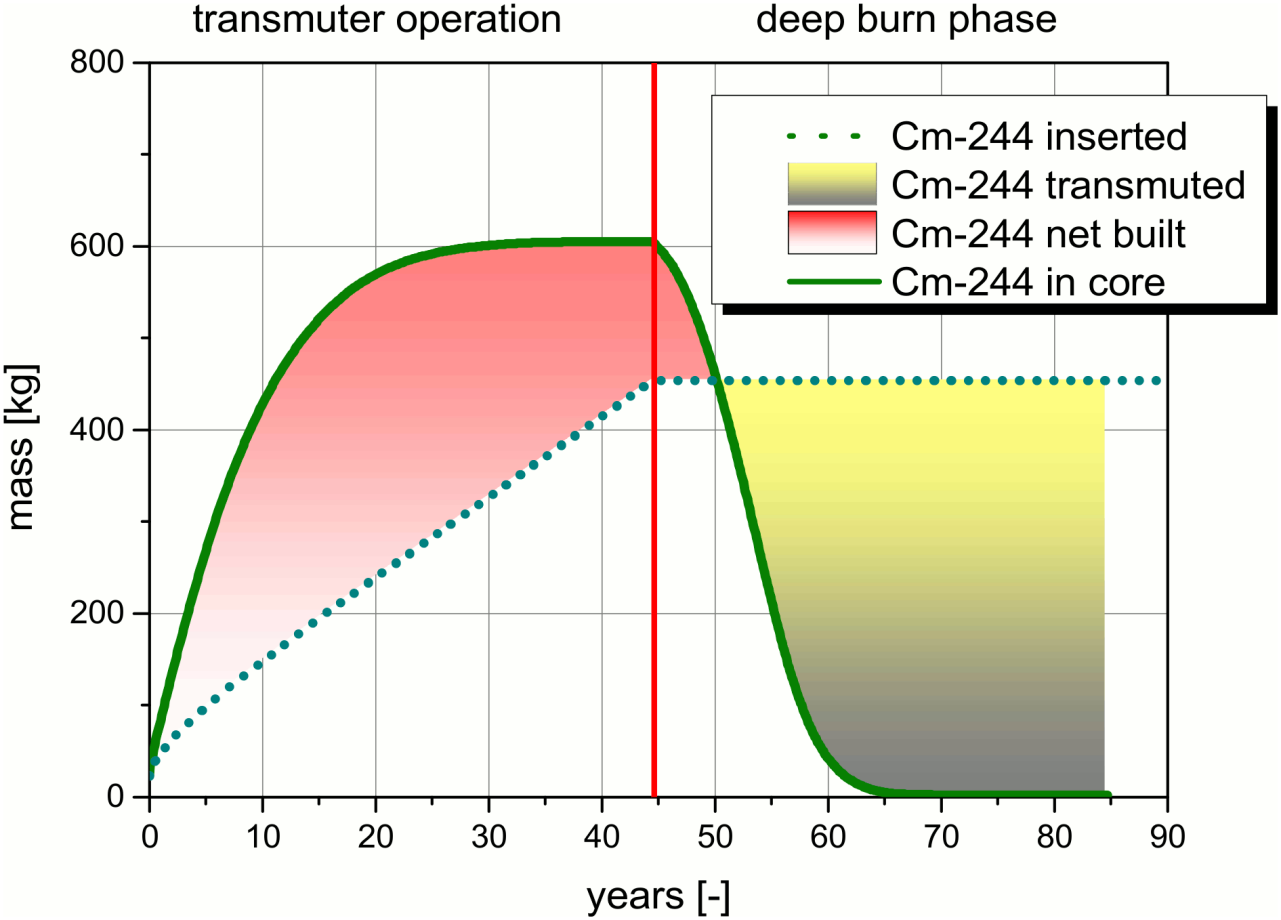
Comparison of the, into the core inserted, Cm-244 mass and the, in the core resident, Cm-244 mass over the operation time.

**Fig 12:**
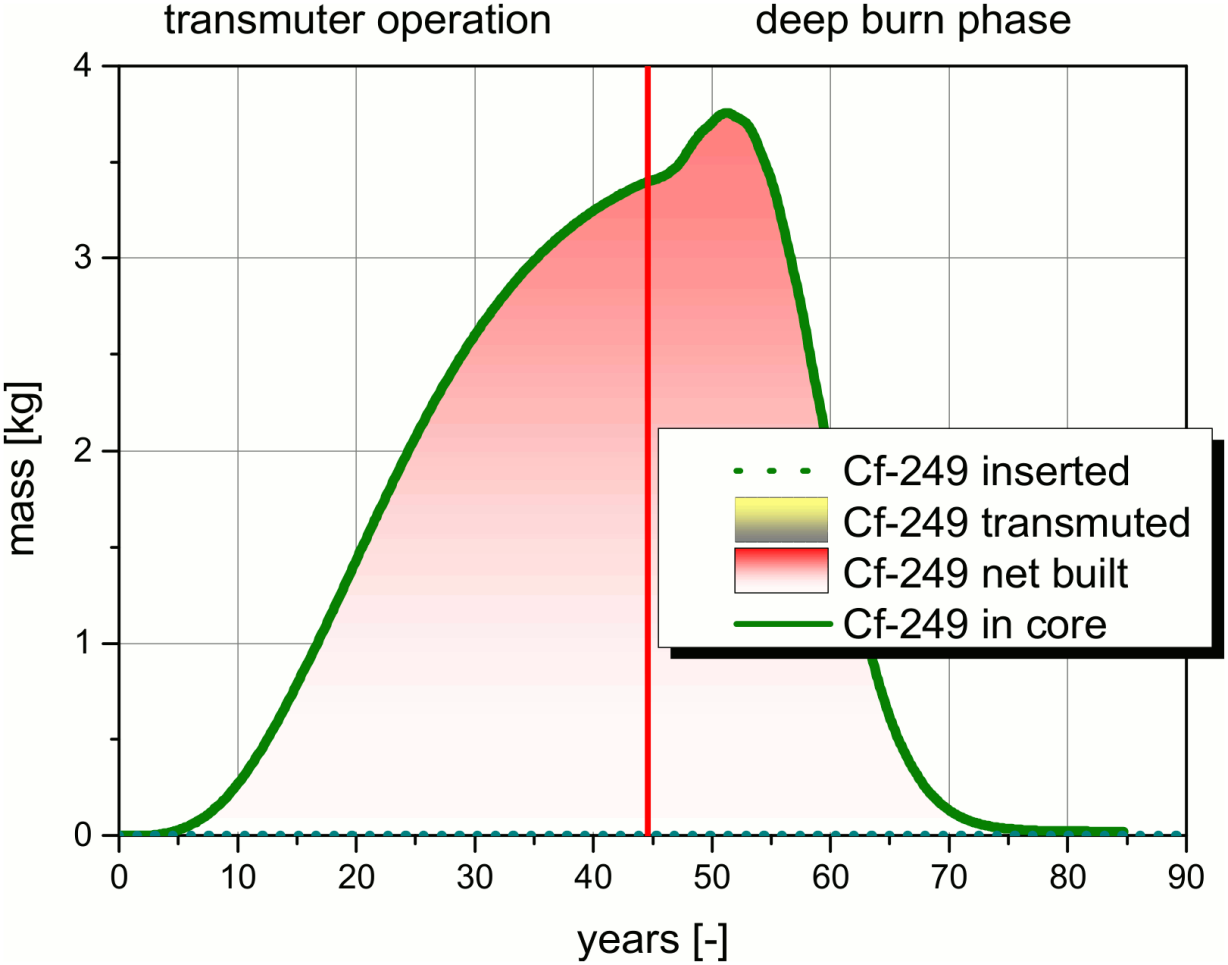
Comparison of the, into the core inserted, Cf-249 mass and the, in the core resident, Cf-249 mass over the operation time.

Transmutation is a process which is generally driven by different nuclear reactions. To illustrate the process, a detailed analysis of the, into the system inserted and the in core resident masses of the, TRUs are given in Figs 9–12. The figures and the detailed discussion are given to deepen the understanding of the processes driving the transmuter operation and the deep burn phase. The major nuclear reactions are the fission reactions and the capture reactions. On the one hand, there are the fission reactions which lead immediately to a reduction of the TRU inventory in the core. On the other hand, there are the capture reactions which lead to breeding processes. In both processes the observed isotope disappears, but as long as no fission takes place the isotope only forms a higher TRU isotope. Thus, the TRU mass is not reduced and often the radiotoxicity of the unwanted higher isotope is even higher than the one of the precursor. Even a net built up of a specific isotope can appear, if the capture processes leading to an isotope predominates over the fission and the capture processes in the special isotope.

The TRU mass inserted into the core are significantly higher than the TRU mass resident in the molten salt in the core over the operation time (see Fig 9). The efficiency of the transmutation after the initial operation period of ~10 years can be clearly observed. In the initial phase, an accumulation of TRUs in the core takes place. However, even in this starting period more TRU is burnt than accumulated. The TRU mass in core is almost constant after ~10 years. This indicates that the TRU amount inserted into the system after this time is immediately burnt in the same cycle, while the TRU content in the salt stays constant. The amount of burnt TRU can be read at each point of the operation as the yellow marked difference only along the y-direction. No TRUs are added in the following deep burn phase anymore. The amount of inserted TRUs stays constant there. However, the TRU mass resident in the core decreases rapidly. Thus, in the deep burn phase, the amount of resident TRUs in the core is eliminated. This can be read from the figure due to the extension of the yellow area down to the x-axis. The accumulated TRUs in the core are transmuted efficiently in a ~15 years lasting deep burn phase. A net built up of TRUs does not appear during the whole operation period neither in the transmuter operation, nor in the deep burn phase. The values at the end of the deep burn phase reflect the mass of burnt material which is given by the distance between the dotted line (inserted TRU) and the solid line (TRU resident in the core). This value has to be compared to the negligible distance between the solid line and the x-axis which reflects the remaining TRUs in the core.

A comparable process can be observed for the Pu-239 mass inserted into the core. The inserted mass is always significantly higher than the Pu-239 mass resident in the molten salt in the core (see Fig 10). This behavior is characteristic for isotopes with a comparably high fission cross section in the fast neutron spectrum. The characteristic for these isotopes is the immediate decrease of the contents from begin of operation on. No accumulation takes place at all, since the inserted amount of these isotopes is immediately burnt in the cycle when they are inserted. Typical representatives are Pu-239, Np-237, and partly Pu-241. The Pu-239 content stabilizes after ~15 years at a value which is lower than the Pu-239 content in the initial TRU vector. This type of isotopes disappears very rapidly at the beginning of the deep burn phase. Both effects can be explained by the high fission cross section. Pu-239 is the main fissile isotope in the starting phase. During the operation, other fissile isotopes are created in breeding processes. A part of the required fissile content is shifted to other isotopes and less Pu-239 is required to sustain the energy production. Finally, the excellent fissile material is fissioned very rapidly in the deep burn phase. New Pu-239 is not bred, since the typical precursor would be U-238 which is almost not available in the core.

The comparison between the Cm-244 mass inserted into the core and the Cm-244 mass resident in the molten salt in the core over the operation time indicates a completely different behavior. This behavior is characteristic for isotopes which are inserted with the TRU loading and which have a comparably low fission cross section in the fast neutron spectrum. Typical representatives are Pu-238, Pu-242, Am-243, Cm-244, and Pu-245. All these isotopes undergo a net built up to an asymptotic value, which can even be higher than the overall inserted amount of the isotope (e. g. in the case of Cm-244 and Cm-245), see Fig 11. Thus, for these isotopes it is possible to find amounts in the salt which are higher than the amount inserted with the feeding material. This is one of the major challenges of the transmutation process. The isotopes are formed by breeding inside the TRU material which is fed into the core to be burnt. The major reason for this behavior is that the isotopic composition of the TRU feed is not the asymptotic one. The asymptotic composition will be formed specific for any kind of reactor and neutron spectrum. In a traditional transmutation scheme ~150 kg Cm-244 would have been created in addition to the already available ~420 kg. This is the situation at the end of the transmuter operation. An interesting fact is that the asymptotic amount is reached after ~30 years. Obviously, the amounts of Cm-244 which are inserted in the following ~15 years are immediately burnt. This effect confirms that Cm-244 can be transmuted. The Cm-244 amount in the core decreases rapidly in the deep burn phase. This is caused by two effects, no new Cm-244 is fed into the core anymore and the amount of precursors for the creation of Cm-244 is decreased. Finally, the Cm-244 will be nearly completely transmuted in ~20 years of deep burn phase. Additionally, the relations between different isotopes should be kept in mind. Pu-239 is given in tons and Cm-244 is given kilograms. There is nearly a factor of 50 more Pu-239 inserted than the maximum mass of Cm-244 in the core.

The very heavy isotopes which are not inserted with the TRU loading are characterized by a comparably low fission cross section in the fast neutron spectrum. These isotopes accumulate during the transmuter operation (see Fig 12). Typical representatives of this group are the higher Cm- isotopes, the Bk- and Cf- isotopes. These isotopes accumulate by breeding processes in the TRU. Breeding processes are more or less unavoidable due to the competition of the fission and capture processes. The formation of Cf-249 starts with a time delay of roughly five years, since the precursor isotopes for the breeding of Cf-249 have to be built first. In the following period, the mass increases to an asymptotic value which is not completely approached in the time of the transmuter operation. The increase of the Cf-249 amount becomes even stronger in the first five years of the deep burn phase. Obviously, the system tends to a new asymptotic value which would be higher for a system with less plutonium. This increase is stopped due to the reduction of breeding which is caused by the reduction of the precursor isotopes for breeding. The time for approaching the maximum value becomes longer, the higher the californium isotope is. However, after 30 years of deep burn phase, all Cf isotopes have almost disappeared.

**Fig 13:**
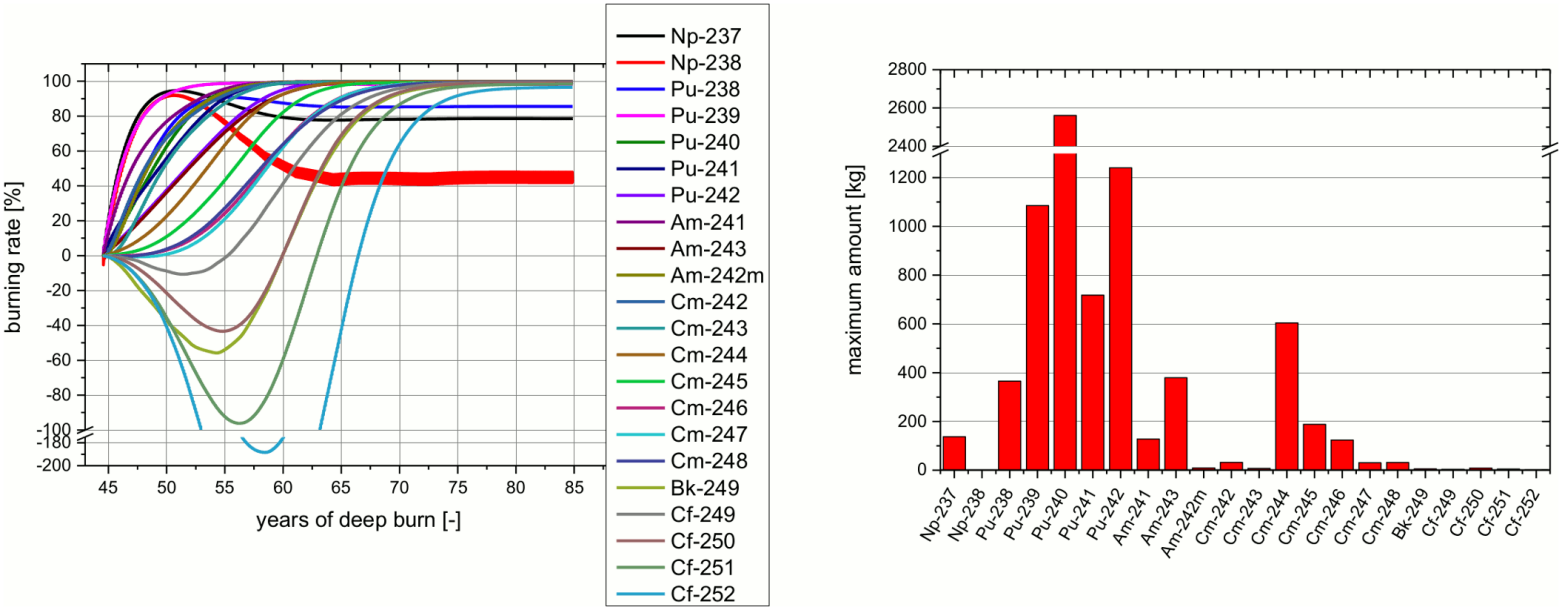
Optimization of the deep burn phase: burning rate for isotopes (left) and maximum amount of isotopes (right).

The different isotopes appearing in the core at the end of the transmuter operation require significantly different time horizons for the burning of in the deep burn phase (see Fig 13, left). This information has to be seen in conjunction with the corresponding maximum amounts of the different isotopes during the deep burn phase (see Fig 13, right). Both parts of the figure form the correlation for the evaluation of the efficiency of the deep burn phase. The left part of the figure helps to understand how much time is required in the deep burn phase to burn each specific isotope. Negative values in the burning rate indicate breeding of an isotope. A process which obviously only appears for Bk and for Cf isotopes. The figure on the right indicates the relevance of these isotopes via the maximum amount of the specific isotope which is appearing during the deep burn phase. There, it is obvious that the Bk and Cf isotopes appear only in a very limited amount during the whole deep burn phase. In comparison it is clear to see, the isotope appearing in the highest mass (Pu-239) is already mostly burnt after only ten years of deep burn. In this way every isotope can be studied. Cm-246 can be identified as the highest isotope which appears in relevant mass (~100 kg). It has almost disappeared after 25 years. Unfortunately, the minimum amount of Np-237 which appears at a comparable mass (~130 kg) lies already at about five years. Thus, a non-optimal amount of Np-237 has to be accepted. A detailed interpretation of the combination of these two figures will help to optimize the number of years of deep burn operation. The operation time depends on the boundary values/conditions which have to be given.

## Conclusions and Outlook

The proposal of the twofold lifecycle in a molten salt reactor has been applied to the specific German situation using an improved code and improved modeling for a neutronic study. Based on the proposal of the twofold lifecycle, it is demonstrated that the transmutation under the objectives of the nuclear phase out decision would be feasible from the neutronic point of view. It is shown that the proposal using a molten salt fast reactor has the potential to burn up to 99.8% (aside the losses in the salt cleanup system) of the TRU currently expected to be accumulated in Germany until the phase out has been executed.

To achieve this goal, three molten salt reactors with 3000 MW_th_ each, as proposed in the EVOL benchmark description, would be required. It has to be mentioned that these reactors rely on a technology which has only been partly demonstrated long time ago in an experimental reactor, up to now. Thus, significant research and development work would be required, see the description below. However, MSFRs have the potential to burn the TRUs efficiently under the specific boundary conditions given in Germany. The advanced reactors would have to be operated for roughly 45 years in a transmuter mode. This operation phase has to be followed by a deep burn mode which has to be optimized. The final burn out of the TRU is strongly dependent on the number of years in the deep burn phase and varies between 97.5% after 10 years and 99.8% after 35 years, aside from possible losses in the salt cleanup system. A salt configuration for the fertile free burning of the TRUs is used which has been proposed in the MOSART concept. The calculations show that the solubility limit for the TRUs is never exceeded during the whole operational life.

A detailed insight into the material balance regarding burning and breeding is given. It demonstrates that already more than 85% of the TRUs can be burnt during the transmuter operation. It has been shown that even isotopes which are accumulated during the transmuter operation can be burnt later on in the deep burn phase almost completely. This fact leads to the request of the optimization of the length of the deep burn phase. The basis for this optimization of the time needed to accomplish the deep burn phase to the requested burn out of TRUs is given. In general, the length of the deep burn phase has to be adapted to the boundary conditions given by the public. Finally, a balance has to be found between the efficient transmutation of all TRUs and the requested operation time of the nuclear reactors. However, the required weighting between these two counteracting requests has to be found in this case in the German public.

Generally, it is demonstrated that transmutation even under the boundary conditions and objectives of the phase out would be possible from neutronic point of view in molten salt fast reactors. Of course, there is still a huge R&D request to bring molten salt fast reactors to application. The major points have already been worked out in the P&T study [7,27] in the described research questions. “Transmutation system dependent research: A safety approach for the evaluation of the design of a reactor system with liquid fuel and integrated fuel processing (salt cleaning) is to be developed. The continuous fission product separation from the fuel and the handling of the fission products is to be improved and optimized. … Nuclear data and reactor physics: Simulation tools for the modeling of the moving fuel, the continuous feeding of fuel, and the fission product removal and release are required for liquid fueled reactors. … Transmutation: The research questions are focused here on all safety relevant aspects and the optimization of the transmutation efficiency of the different systems. Thermodynamic, fuel behavior, material research, and technological development (models, laboratory scale experiments and full scale component tests) are required for the safe operation of the coolant/fuel of a transmutation facility. Extensive R&D is required and has to be backed with irradiation tests for the fuel as well as for the structural materials.”

It has to be mentioned that this R&D work should be carried out not only in one country but in a European or even better world wide co-operation. The problem of the Pu or even better TRU accumulation as consequence of the extensive light water reactor operation appears in several countries. Especially, the problem arises in all nations following strategies which do not envisage an extensive use of the fast reactor technology for energy production. All these countries together should stimulate an international endeavor to solve the TRU accumulation problem. The problems of the plutonium accumulation and the long term consequences have already been described in the IAEA bulletin in 1998. “Within 200 years, the protection afforded by intense radioactivity (spent fuel standard) will disappear as the result of the decay of most radioactive nuclides. If the spent fuel is buried in a geological formation, it might be regarded as a potential ‘plutonium mine’ meaning that at some later point in time the buried plutonium could be mined and extracted”. [41] A comparable discussion took place during the German P&T study and led to the formulation of one of the chances of P&T (“The application of P&T on industrial level has the potential for a plutonium content in the final disposal which is negligible after the application of P&T. Thus, there is no risk of misuse and theft of Plutonium (from the final disposal), anymore” [7, 8].) Both excerpts indicate that it is time to have a closer look to the Plutonium management in the future.
